# Coordinated care affects hospitalization and prognosis in amyotrophic lateral sclerosis: a cohort study

**DOI:** 10.1186/s12913-015-0810-7

**Published:** 2015-04-02

**Authors:** Valérie Cordesse, Florence Sidorok, Priscilla Schimmel, Josiane Holstein, Vincent Meininger

**Affiliations:** ALS Community Network, Hôpital de la Salpêtrière, 47 Boulevard de l’Hôpital, Paris, 75013 France; Medical information Department, Hôpital St Antoine, Paris, France

**Keywords:** Amyotrophic lateral sclerosis, Intensive care, Cohort study, Coordination of care

## Abstract

**Background:**

To determine whether an integrated approach to coordination of care influences hospitalization and clinical outcomes in a chronic neurological disease, amyotrophic lateral sclerosis.

**Methods:**

We followed up 2452 patients with probable or definite amyotrophic lateral sclerosis from 2000 to 2012. Two cohorts were compared before and after the creation of a community care network for this disease in Ile de France in 2006. During these two periods, the medical and paramedical care teams and formal standards of care were identical; the only difference was the coordination by the network. To investigate hospital and emergency department use, we used number of patients, number of stays, and number of days. For clinical outcomes, we used slopes of functional deterioration, and Kaplan–Meier and Cox models for survival.

**Results:**

All hospitalization variables decreased after the creation of the network, which was not explained by admissions elsewhere. The slope of functional deterioration was significantly different before (1.03 ± 1.57 points/month) and after (0.79 ± 0.80 points/month; p = 0.002) creation of the network. Patients included in the network had a median survival time of 13.2 months more (log rank test; p < 0.001). In the Cox model, the network intervention was associated with a 45% decrease in relative risk of death during the period of the study (p < 0.001).

**Conclusions:**

Network care was associated with fewer hospital admissions, reduced functional deterioration and later mortality in ALS. These results suggest that proactive coordination between carers in chronic and complex diseases could have a positive impact on hospitalization and the clinical course of the disease.

**Electronic supplementary material:**

The online version of this article (doi:10.1186/s12913-015-0810-7) contains supplementary material, which is available to authorized users.

## Background

An important challenge for health systems is patients with complex diseases requiring care from multiple healthcare professionals over time. This has given rise to the concept of multidisciplinary care in chronic diseases, including neurological diseases [1-3].

Multidisciplinary care is only one aspect of team healthcare [4] and has no single pattern of operation [5]. However, continuity of information is especially important in improving team performance [6,7]. Team coordination can reduce the negative consequences of silo decision-making, where different carers independently recommend different care strategies with a negative impact on continuity of care, particularly when the patient returns home.

To improve team healthcare, we set up a 2-year university course to train care pathway coordinators in coordinating other healthcare professionals (Additional file 1). To evaluate the impact of these new professionals, we focused on one chronic neurological disease affecting all aspects of health, amyotrophic lateral sclerosis (ALS). Since 2000 (see details in Additional file 1), nearly all ALS patients in the Ile de France region (IDF) have been followed up by the same single team following the same multidisciplinary care protocol. This team was based at a dedicated specialized center at Salpêtrière Hospital, Paris. Since 2005, a team of care pathway coordinators based in a community care network has worked alongside the Salpêtrière team.

To analyze their impact, we compared outcomes in two groups of ALS patients: those seen before October 2005 (patients before network: PBN group) and those seen after that (patients in network: PIN group).

## Methods

### Data sources and study population

For hospital admissions at Salpêtrière Hospital, the data used came from the Programme Médicalisé des Systèmes d’Information (PMSI), which systematically records all data during each hospital admission.

For clinical impact of coordination, we used the medical data from our database and we compared the data of the PIN group since 1st October 2005 with data of the PBN group followed up between 1st January 2000 and 1st October 2005. Patients from the PBN group who entered the network were excluded from the study irrespective of their date of entry, which did not affect the analyses (Additional file 2). We took into account only “incident” patients, i.e., patients whose diagnosis was definitely established at their time of enrollment in the center (PBN) or in the network (PIN). This study was approved by the Ethical Committee of Salpêtrière Hospital, which agreed that consent was not necessary under French law. A similar proportion of patients in each group (8.9% in the PBN, 8.3% in the PIN) participated in clinical trials during their follow up. All patients had a diagnosis of possible, probable or definite ALS according to the El Escorial criteria [8]. For the survival analysis, date of death was confirmed by the treating physician, the family and, if necessary, the death certificate from the town hall.

To evaluate the number of ALS patients in IDF, we used the SNIRAM (Système National Inter Régime d’Assurance Maladie) database, available since 2012, which collects all medical information for patients. In France, all ALS patients are given riluzole (*Rilutek*®), so monitoring riluzole consumption gives almost complete data on the number of patients with ALS.

### Clinical measures and functional outcomes

The following data were collected: age, sex, site and date of onset, delay since onset of the disease [9], date of death, date of gastrostomy, date of non-invasive ventilation and riluzole consumption.

The progression rate of function at first visit (ΔFS) [10] was calculated. Since the ALSFRS-R decline is linear [11], we used the PIN group to compare the slope of the ALSFRS-R scale before the introduction of coordinated care with the slope during follow-up by the care pathway coordinator.

### Hospitalization data

For admissions to Salpêtrière Hospital, we analyzed the number of patients hospitalized for more than 24 hours, the number of stays (each stay was counted, even if a patient came back several times) and the number of days in hospital.

### Statistical analysis

Quantitative variables were compared by analysis of variance (ANOVA) and qualitative variables using Pearson’s chi-squared test. Quantitative variables were dichotomized. The cut-offs used were 60 years for age and 1.09/month for the slope at entry of the ALSFRS-R.

For the survival analyses in the PIN group, we retained patients included between 1st October 2005 and 31st December 2008 to avoid biasing the survival data by patients with insufficient follow-up. For the surviving patients, date of censoring was 1st October 2013 for the PIN group and 1st January 2008 for the PBN group to avoid an imbalance in the observed follow-up period between the two groups (i.e., a maximum of 8 years for both groups).

The survival curves for the PIN and PBN groups were compared using the Mantel Cox log-rank test. Prognostic factors for survival were assessed using the Cox proportional hazard method, by entering into the model dichotomous variables: participation in the network, age at disease onset, site of disease onset, gender, ΔFS, NIV (non-invasive ventilation) and gastrostomy. The possible role of cognitive impairment as a prognostic factor was not used considering the lack of certainty on its role [12].

All significance levels were two-sided with a probability threshold of p < 0.05. All statistical analyses were carried out with SPSS version 11 · 0.

## Results

### Effect of coordinated care on hospital admissions

From 2000 to 2012, 2452 patients were followed up, with 188 (±16) patients enrolled per year. The mean enrollment rate was similar between the two groups (Table 1). In 2012, 623 patients were given riluzole in the IDF (SNIRAM database) and the network monitored 570 patients, i.e., 91.5% of patients with ALS in the IDF. Table 1 shows all variables for hospitalization decreasing over time after the initiation of the coordinated care. The proportion of patients admitted to Salpêtrière compared with other hospitals remained stable at around 91%, confirming that there was no reallocation of patients to other hospitals in Ile de France.

**Table 1:** **Data for hospitalization, incidence and prevalence**

**Year**	**2000**	**2001**	**2002**	**2003**	**2004**	**2005**	**2006**	**2007**	**2008**	**2009**	**2010**	**2011**	**2012**
Total number of patients followed up	413	418	413	457	418	414	433	457	514	543	559	572	570
Absolute number of patients admitted	328	354	367	422	358	222	181	202	193	198	216	192	216
Proportion of patients admitted (1)	79,42%	84,69%	88,86%	92,34%	85,65%	53,62%	41,80%	44,20%	37,55%	36,46%	38,64%	33,57%	37,89%
Annual number of stays (2)	469	498	566	581	546	290	209	232	220	236	261	231	266
Rehospitalization frequency index (3)	1,43	1,41	1,43	1,38	1,53	1,31	1,15	1,15	1,14	1,19	1,21	1,20	1,23
Risk of admission per patient index (4)	1,11	1,19	1,37	1,27	1,28	0,70	0,51	0,62	0,38	0,31	0,35	0,32	0,36
Ratio/emergency (5)	7,33%	7,18%	7,99%	4,60%	6,07%	6,28%	6,62%	6,67%	2,97%	3,75%	3,78%	4,14%	4,28%

^1^- Ratio of number of patients admitted during 1 year/number of patients followed up in the same year by the network.

^2^- Total number of hospitalizations or stays, each stay was counted, even if a patient came back several times.

^3^ -Ratio of hospital stays to the number of patients admitted to hospital.

^4^- Ratio of hospital stays to the total number of patients followed up.

^5^- Ratio of patients admitted for uncontrolled emergency situations to the total number of patients followed up.

### Effect of coordinated care on interventions

We assessed the impact of the coordinated care on the main therapeutic interventions in ALS [13]. All patients were treated with riluzole. For gastrostomy, both the frequency (9% of patients per year) and the mean interval between disease onset and gastrostomy (PIN 32.0 ± 27.1 months vs PBN 28.5 ± 15.2 months; p = 0.22) were not modified by the introduction of the coordinated care. Since 2002, 584 patients received NIV, with a regular increase in the proportion of ventilated patients/year from 8.1% in 2002 to 36.3% in 2012. Other formal standards of care regarding concomitant medications (including use of antibiotics) remained strictly identical from 2000 to 2012. For administrative reasons, use of cough assistance or high frequency chest wall oscillations was not allowed until 2013 and was not used by the patients in our study. In summary, from 2000 to 2012 the medical management of patients was not formally modified, except for NIV.

### Effect of coordinated care on survival and rate of functional deterioration

To study the effect of the coordinated care on functional deterioration, we used the PIN group to compare the slope before the inclusion of patients in the coordinated process and that during their subsequent follow-up (n = 506). There was a significant difference in average slope before entry to the network and during the follow-up by the network (1.03 ± 1.57 points/month before the network; 0.79 ± 0.80 points/month during; p = 0.002).

**Figure 1:**
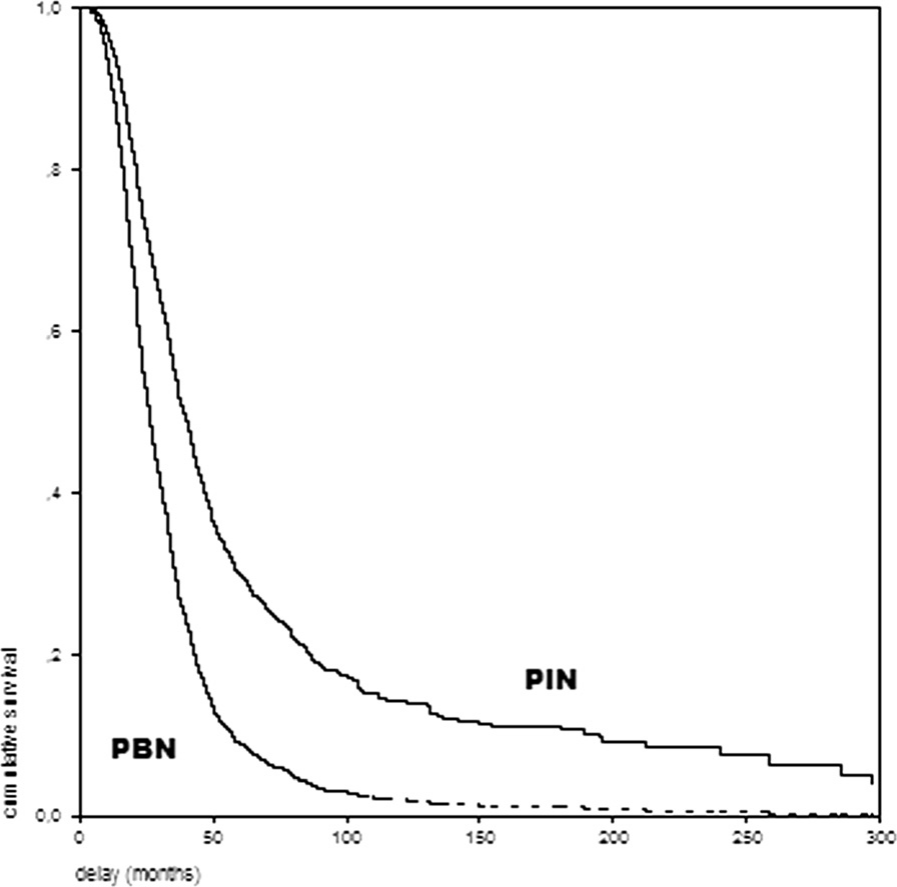
**Kaplan–Meier plots of survival in ALS patients before (PBN) and after (PIN) coordinated care introduction.**

To establish the effect of the coordinated care on survival, two groups of patients (PIN and PBN) were compared. These two groups were balanced for the most powerful prognostic variables (Table 2). There was a significant difference in survival curves for the PIN group compared with the PBN group (log rank test; p < 0.001; median survival 38.8 months for the PIN group vs 25.6 months for the PBN group) (Figure 1).

**Table 2:** **Demographic characteristics of ALS patients before the network (PBN) and in the network (PIN)**

**Variable (mean +/− SD)**	**Patients before network**	**Patients in network**
Age (year)	62 · 6 (11 · 7)	61 · 2 (12 · 9)
Sex (% male)	52.8%	54.8%
Site of onset (bulbar/spinal) %	33/67	34 · 4/65 · 6
Disease duration (month) before enrollment	21.8 (19.2)	21.5 (17.8)
ΔFS (/month) at entry mean (SD)	0 · 97 (0 · 78)	0 · 94 (1 · 60)

Univariate analysis showed a significant association with survival for age, gender, site of onset, initial slope of deterioration, NIV, gastrostomy and coordinated care (Hazard ratio for care = 0.502 [95% CI: 0.439–0.573; p < 0.001]). In a multivariate analysis (Table 3), the model retained only site of onset, initial slope of deterioration and the coordinated care. Age, gender, NIV, and gastrostomy were not retained. Sensitivity analyses were conducted to clarify the possible role of long survivors and NIV (Additional file 2).

**Table 3:** **Survival analysis adjusted for prognostic factors (Cox model)**

	**HR**	**95% CI for HR**	**p**
Age	0 · 845	0 · 649–1 · 029	0 · 094
Sex	0 · 915	0 · 748–1 · 118	0 · 384
Site of disease onset	1 · 789	1 · 433–2 · 235	0 · 001
Slope deterioration	0 · 326	0 · 261–0 · 406	0 · 001
NIV	1 · 251	0 · 953–1 · 642	0 · 106
Gastrostomy	0 · 985	0 · 775–1 · 252	0 · 901
Coordinated care (PBN/PIN)	0 · 549	0 · 439–0 · 687	0 · 001

Legend: All variables are dichotomized. The cut-offs used were 60 years for age and 1 · 09/month for the slope at entry of the ALSFRS-R (ratio 2/3, 1/3). For disease onset, 0 was bulbar, 1 was spinal.

## Discussion

Our study compared two types of care management: a multidisciplinary approach carried out at the hospital only and a coordinated care between home and hospital by trained professionals. Our results show that the coordinated care decreases the number of hospital admissions and the rate of functional deterioration and appears to delay mortality.

Our results showed a marked decrease in hospital admissions with the initiation of the coordinated care. This decrease was not due to a reallocation of patients, nor to longer hospital stays because the mean annual number of days of hospitalization remained stable at 3.3 days (Table 1). There are several possible explanations for this impact on hospital admissions. An interaction with a change in care trends over time [14] is unlikely since both the paramedical and medical care teams remained strictly identical from 2000 to 2012. Due to this stability, all the standards of care, including concomitant medications and nutritional recommendations, and protocols for care both in and out of hospital, and for home health/hospice care remained strictly identical during the two periods of comparison. The possible role of clinical trials was not retained since the proportion of patients included in these trials was identical in the two groups.

Given this stability, the better outcomes observed since 2006 are likely to be a direct consequence of the care management, as previously suggested [15]. The better scheduling of care reduces the need for emergency admissions. It reduces the multiple concurrent medical issues, which are major drivers of hospital admission [16]. The coordination of care allows personalised care coordination integrating all medical, psychological and social aspects of management over the entire disease duration. The coordinators provide a tight link between community-based carers and the hospital. Furthermore, their follow-up at home by regular telephone calls help verify adherence to medical recommendations. This coordination improves adherence to recommendations and avoids breaks in the continuity of care, and potential sources of difficulties for patients and carers [7].

Changes over time in the population studied can be another source of bias. All characteristics of our patients (Table 2) were stable and comparable to those previously reported [16]. Given the data of SNIRAM, it appears that nearly 92% of ALS patients in Ile de France were included in the study, making selection bias unlikely.

We observed a relationship between the coordinated care and health status by slowing down the rate of functional deterioration and by improving survival time. For both cohorts, we used the same HAS guidelines [13] for care management. Even if not randomized, our study compared two groups of patients with otherwise strictly identical care management. Unidentified prognostic factors could explain the difference in survival, but the two groups were comparable for the main ALS prognostic factors. The possible role of cognitive impairment as a prognostic factor was not retained because of the lack of certainty on its role [12].

Only two care variables differed: the coordinated care and the more frequent use of NIV. Using the Cox model adjusted for the most significant variables in ALS [17], the univariate analysis shows that both coordinated care and NIV influence the survival outcome. In the multivariate analysis, coordinated care had a stronger impact, although this does not mean that it is solely responsible for the improved outcome. Sensitivity analyses strengthen this conclusion (Additional file 2). We previously reported an improvement in survival times since 2005 [18], and we suggested that NIV might play a role in this. However, we were not able to provide quantitative data on ventilation and we did not take into account the role of the coordinated care. This study now confirms our previous conclusions on the improvement of survival times and the role of NIV but adds new information on the role of coordination of care.

The impact of multidisciplinary care on disease evolution in ALS has been disputed [19]. A recent study [20] concluded that case management in addition to multidisciplinary care in ALS did not improve health-related quality of life or modify disease progression. However, the outcomes were different, there were missing data and the paper did not provide clear data on the real involvement of the case managers.

Multidisciplinary care has been an important, but incomplete step in care improvement. Other disappointing experiences were proposed such as the “patient-centered medical home” in which a medical practice actively managed patients’ chronic conditions [21]. A possible reason for such disappointing results, including in ALS [20], could be the need for primary care practices to have a strong system of care managers [22]. In our study, rating the intensity of the management [23] showed that the coordinators reached 16 criteria out of 18, which favors high intensity management. Our results indicate that such an intense system of care management by care managers intervening between the hospital and the community and between healthcare professionals, patients and carers had a favorable effect on both hospital admissions and prognosis in a specific model of chronic disease. The confirmation of these conclusions by further studies could have a major impact on national health system strategies and implementation of new jobs in health care.

## Conclusions

In this study, network care was associated with fewer hospital admissions, reduced functional deterioration and later mortality in ALS. These results suggest that compared to a multidisciplinary care a proactive coordination between carers in chronic and complex diseases could have a positive impact on hospitalization and the clinical course of the disease.

## Additional files

Additional file 1:
**Organization of the health pathway of the ALS patients in Ile de France.** Role of the care pathway coordinators. Additional file 2:
**Sensitivity analyses.**
