# Supplementary material for: Coordinated care affects hospitalization and prognosis in amyotrophic lateral sclerosis: a cohort study
Source: BMC Health Serv Res. 2015 Apr 2;15:134. doi: 10.1186/s12913-015-0810-7 (PMC4384378; doi:10.1186/s12913-015-0810-7)
Supplement: Additional file 1: — Organization of the health pathway of the ALS patients in Ile de France. Role of the care pathway coordinators. [file 12913_2015_810_MOESM1_ESM.doc]

Additional file 1

**Organization of the health pathway of the ALS patients in Ile de France. Role of the care pathway coordinators.**

Since 2000, Salpêtrière has had a dedicated center with five neurologists, one pneumologist, one gastroenterologist, two speech therapists, one physiotherapist, two specialized nurses, one dietician, and three social workers. This center is recognized as the national referral center for ALS by the Ministry of Health. The professionals in the center receive ALS out-patients every 3 months for a 1-day multidisciplinary evaluation. At the end of the day, they provide a synthesis document with recommendations. For nearly all patients (more than 95%), diagnosis of ALS is given at the center by a neurologist who will be in charge of the follow up. The referral center also provides in-patient care for gastrostomy, non-invasive ventilation (NIV), or palliative interventions at the Department of Nervous System diseases or at the Department of Respiratory diseases, both located at Salpêtrière. The Department of Gastroenterology is responsible for percutaneous endoscopic gastrostomy (PEG) interventions. Of the medical and paramedical staff at the referral center, only the physiotherapist has changed since 2000.

Since October 2005, there is a community care network in support of the referral center for ALS patients. In the community care network, there are four coordinators of care, one psychologist, and one physiotherapist. The coordinators only are trained during a 2-year university course at Université Pierre et Marie Curie [1]. The psychologist gives punctual advices under request either of the neurologists at the Center or of the coordinator. The physiotherapist is part time (1/4) and intervenes only on the request of the coordinators to contact the physiotherapist at home and explain how to manage the physiotherapy.

The national referral center has been evaluated twice [2,3] by the Ministry of Health. Both reports underlined the importance of the relations between the center and the community care network. They also underlined the very good evaluations by the patients, relatives and care givers of the actions of the center and of the network and the complementary services provided.

These two structures are tightly linked but independent. The health network’s care coordinators are responsible for coordinating care-givers and providing a link between the center and patient’s home. A few days after the initial consultation for diagnosis by a neurologist at the center, there is a follow-up consultation with the care coordinators, during which the disease and its care are further explained. At the end of this consultation, the coordinator gives a medical dossier to the patient. This will act as a medical record as the disease progresses, and is a link between care-givers. The roles of the coordinators [1] are:

- To evaluate all the needs of the patients and relatives in all domains (medical, social and others) by regular phone calls (every 4–6 weeks) or by phone calls at the initiative of the patients and/or relatives;

- To ensure (by systematic phone calls to GP, other care-givers and the patients and relatives) the correct follow-up of all recommendations made by the center (after a multidisciplinary clinic or after a hospitalization) and their constant adjustment with time;

- To regulate all medical and social interventions particularly during emergency situations and/or social crisis. In these situations, the care pathway coordinators make the initial check up by phone and decide on the most appropriate decisions. In case of orientation in an emergency department, they are in charge of transmitting all medical information and have to ensure a constant follow-up of the decisions thereafter.

- To inform (by phone, or by mail) all healthcare professionals who have contact with the patient at home about recommendations made by the center and to ensure that these recommendations have been clearly understood and implemented;

- To train the care-givers at regular intervals to explain the disease and the care programs as soon as a patient needs new professionals involved.

- To communicate about the disease and make appropriate links with social structures to ensure a quick adaptation of social support during the course of the disease.

Most of these interventions are performed by phone calls. However, when necessary the coordinators intervene directly at home.

Compared with multidisciplinary care, the coordinated care by the network allows personalized care coordination, integrating all medical and social aspects of management over the entire disease duration. This coordination avoids breaks in the continuity of care, a potential source of difficulty for patients and carers.

REFERENCES

1. Meininger V, Fontaine B, Grabli D, Basdevant A, Clement K, Vignot M, Cordesse V: [**What about a new job in the health system: The coordinator of care**]. *Presse Med* 2014, Jul 26. pii: S0755-4982(14)00351-0. doi: 10.1016/j.lpm.2014.06.006.

2. Corcia P, Bourdillon F. **Evaluation du centre de référence national de la prise en charge de la sclérose latérale amyotrophique (SLA) de Paris**. 2008. <http://www.sfsp.fr/publications/fichiers/rapportSLA.pdf>

3. **Rapport Evaluation des dispositifs mucoviscidose et sclérose latérale amyotrophique**. 2009. <http://fulltext.bdsp.ehesp.fr/Ministere/Dhos/Publications/2009/ISO20035671-1.pdf>
