# Supplementary material for: Coordinated care affects hospitalization and prognosis in amyotrophic lateral sclerosis: a cohort study
Source: BMC Health Serv Res. 2015 Apr 2;15:134. doi: 10.1186/s12913-015-0810-7 (PMC4384378; doi:10.1186/s12913-015-0810-7)
Supplement: Additional file 2: — Sensitivity analyses. [file 12913_2015_810_MOESM2_ESM.doc]

Additional file 2

**Sensitivity analyses**

Two types of sensitivity analyses were performed.

1) As stated in the Materials and Methods section, we excluded from the study patients from the PBN who entered the network irrespective of their date of entry.

As these patients were long survivors, we conducted a sensitivity analysis including them.

The results were strictly identical. In univariate, the coordinated care remained significant (p < 0.001) (HR = 0.546 [95% CI: 0.498–0.598]). In the multivariate analysis, NIV remained excluded (HR: to 0.895 [95% CI: 0.760–1.053]) and coordinated care remained significant (HR: 0.535 [95% CI: 0.466–0.614]).

2) To obtain information on the possible interrelation between the two variables, coordinated care and NIV, we performed two sensitivity analyses: one to strengthen the role of NIV, one to suppress it. To strengthen the role of NIV, we analyzed the consequence of censoring all patients with NIV at the date of initiation of the NIV. The coordinated care remained significant both in univariate (p < 0.001) (HR = 0.436 [95% CI: 0.389–0.489]) and multivariate analyses (p < 0.001) (HR = 0.485 [95% CI: 0.403–0.583]). The site of onset and the slope of deterioration remained selected in the model, but NIV remained excluded.
To further strengthen the role of NIV, we analyzed the consequence of considering all patients with NIV as alive either at the date of their death or at the date of censoring. The coordinated care remained significant both in univariate (p < 0.001) (HR = 0.391 [95% CI: 0.349–0.439]) and multivariate analyses (p < 0.001) (HR = 0.420 [95% CI: 0.339–0.520]). The site of onset and the slope of deterioration remained selected in the model, but NIV remained excluded.
In another analysis, we considered all patients with NIV deceased at date of initiation of NIV. The coordinated care remained significant in both univariate (p < 0.001) (HR = 0.675 [95% CI: 0.611–0.746]) and multivariate analyses (p < 0.001) (HR = 0.635 [95% CI: 0.545–0.740]).
